# The Analysis of PTPN6 for Bladder Cancer: An Exploratory Study Based on TCGA

**DOI:** 10.1155/2020/4312629

**Published:** 2020-05-13

**Authors:** Chengquan Shen, Jing Liu, Jirong Wang, Xiaokun Yang, Haitao Niu, Yonghua Wang

**Affiliations:** ^1^Department of Urology, The Affiliated Hospital of Qingdao University, Qingdao, Shandong, China; ^2^Department of Research Management and International Cooperation, The Affiliated Hospital of Qingdao University, Qingdao, Shandong, China

## Abstract

*PTPN6* (protein tyrosine phosphatase nonreceptor type 6), a tyrosine phosphatase, is known to be signaling molecules that regulate a variety of cellular processes including cell growth, differentiation, mitotic cycle, and oncogenic transformation. Previous studies have demonstrated that *PTPN6* expression is relatively elevated in several malignancies. However, the role of *PTPN6* in bladder cancer (BC) remains unclear. The purpose of this study was to explore the prognostic value of *PTPN6* in BC. RNA-seq data from The Cancer Genome Atlas (TCGA) was used to identify the expression level of *PTPN6* in BC. The relationship between clinical pathologic features and *PTPN6* were analyzed with the Wilcoxon signed-rank test. The prognostic and predictive value of *PTPN6* was evaluated by survival analysis and nomogram. Gene Set Enrichment Analysis (GSEA) was conducted to explore the potential molecular mechanisms of *PTPN6* in BC. Finally, Tumor Immune Estimation Resource (TIMER) was applied to investigate the relationship between *PTPN6* and immune cell infiltration in the tumor microenvironment. Results indicated that *PTPN6* was overexpressed in BC tissues compared with normal bladder tissues and was significantly correlated with grade, stage, T, and N. Survival analysis showed that low expression of *PTPN6* was significantly related to the poor overall survival (OS) in BC patients. Coexpression analysis showed that *PTPN6* and *TNFRSF14* (Tumor necrosis factor receptor superfamily member 14) have a close correlation in BC. GSEA showed that multiple cancer-associated signaling pathways are differentially enriched in the *PTPN6* high expression phenotype. Moreover, the expression level of *PTPN6* was positively associated with the infiltration of B cells, CD4+T cells, dendritic cells, and neutrophils and negatively associated with CD8+ T cells and macrophages in BC. In conclusion, we identified that *PTPN6* may be a novel prognostic biomarker in BC based on the TCGA database. Further clinical trials are needed to confirm our observations and mechanisms underlying the prognostic value of *PTPN6* in BC also deserve further experimental exploration.

## 1. Introduction

Bladder cancer (BC) is the most common malignant neoplasm in the urinary system [1]. Over the past decade, significant progress has been made regarding the mechanisms, diagnosis, and therapy of BC [2]. Nonetheless, the high recurrence rate of nonmuscle invasive BC and the poor prognosis of advanced BC are still the main obstacles to the treatment of BC. At present, the mechanism of BC research remains poorly understand, but genetic, epigenetics, and environmental factors are certainly involved in the tumorigenesis and progression of BC. Over the past few years, the advances in genomic methods have expanded our knowledge about gene expression, genetic, and epigenetic alterations at the pan-genomic level in various malignancies. Genomic studies led to the identification of tumor subgroups that have distinct biology and variable prognosis allowing for the development of prognostic molecular markers [3]. *PTPN6* is a nonreceptor protein tyrosine phosphatase that can act as a tumor suppressor by dephosphorylating oncogenic kinases [4]. Previous studies indicated that *PTPN6* was associated with the prognosis and progression of cancers, such as hepatocellular carcinoma, renal cell carcinoma, and gastric cancer [5–7]. Moreover, *PTPN6* can enhance the efficacy of chemotherapeutic and can combine with blocking antibodies in cancer immunotherapy [4, 8]. However, the correlation between *PTPN6* and the prognosis of BC remains unclear.

In the present study, we identified the prognostic value of *PTPN6* expression in BC based on the TCGA database. In addition, we explored the potential molecular mechanisms of *PTPN6* in BC and the relationship between *PTPN6* and immune cell infiltration in the microenvironment of BC.

## 2. Materials and Methods

### 2.1. Data Collection

The gene expression profiles and corresponding clinical information of BC patients were obtained from TCGA official website (http://tcga-data.nci.nih.gov/tcga/). Four hundred and twelve BC patients were enrolled in our study, and detailed clinical information is shown in Table 1.

### 2.2. Statistical Analysis

Wilcoxon signed-rank test was used to identify the expression level of *PTPN6* between BC and normal samples and analyze the relationship between *PTPN6* and clinicopathologic characteristics in BC. Then, we divided BC patients into high expression and low expression groups according to the lower quartile (Q1), median, and upper quartile (Q3) values of *PTPN6*. Kaplan-Meier curves were plotted to evaluate the significant difference in OS between high expression and low expression groups. Univariate and multivariate Cox analyses were used to compare the effect of *PTPN6* expression on survival along with other clinical characteristics.

### 2.3. Identification of *PTPN6* Coexpression Genes and Construction of a Prognostic Nomogram

We apply cBioportal (https://www.cbioportal.org/), an online tool based on the TCGA database, to identify sets of coexpression genes and select the most relevant gene for *PTPN6* according to the *P* value. Then, clinical factors (age, gender, stage, T, M, and N) and genes expression levels were used to construct a prognostic nomogram to evaluate the probability of 1-, 2-, and 3-year OS for BC patients via the R package (https://cran.r-project.org/web/packages/rms/) [9].

### 2.4. Gene Set Enrichment Analysis

GESA is a computational method that determines whether an a priori defined sets of genes show statistically significant, concordant difference between two biological states [10]. In this study, GSEA firstly generated an ordered gene list according to the correlation between all genes and *PTPN6* expression, and then elucidated the significant survival difference observed between high expression and low expression groups. Gene set permutations were performed 1000 times for each analysis. The expression level of *PTPN6* was used as a phenotype label. The nominal *P* value and normalized enrichment score (NES) were used to sort the pathways enriched in each phenotype.

### 2.5. Correlation Analysis between *PTPN6* Expression and Immune Cell Infiltration in BC

*PTPN6*, as an immune-related gene, has been demonstrated that influence the prognosis of cancer by regulating the immune progress and remodeling the immune microenvironment of a tumor. Thus, we employed Tumor Immune Estimation Resource (TIMER), a useful resource for comprehensive analysis of tumor-infiltrating immune cells, to investigate the relationships between *PTPN6* expression and immune cell infiltration [11]. TIMER algorithm allows users to estimate the composition of six tumor-infiltrating immune cells subsets (B cells, CD4+ T cells, CD8+ T cells, macrophages, neutrophils, and dendritic cells). The immune cells infiltration levels of BC patients were derived from the TIMER website, and the correlation between the *PTPN6* expression and six tumor-infiltrating immune cells was conducted in R.

## 3. Results

### 3.1. Patients Characteristics

As shown in Table 1, 412 primary tumors with both clinical and gene expression data were downloaded from the TCGA database. The median age at diagnosis of patients was 69 years old. The gender included 108 females and 304 males. The histologic grade distribution of BC included high grade and low grade, 94.17% of the tumors were high grade, and 5.1% were low grade. Stage I disease was found in 2 patients (0.49%), stage II in 131 (31.8%), stage III in 141 (34.22%), and stage IV in 136 (33.01%). T0 was found 1 patient (0.24%), T1 in 3 (0.73%), T2 in 120 (29.13), T3 in 196 (47.57%), and T4 in 59 (14.32%). 21 of 412 (5.1%) cases had distant metastases. 127 of 412 cases (30.82%) had lymph node metastases.

### 3.2. *PTPN6* Was Associated with the Prognosis of BC

**Figure 1:**
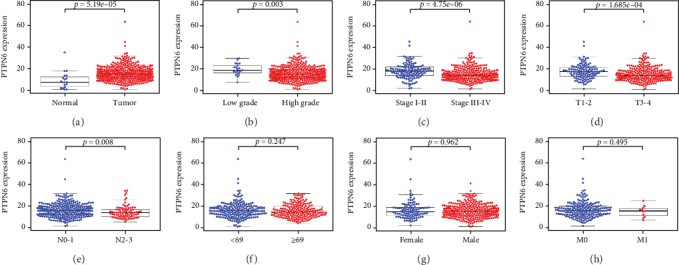
The expression level of *PTPN6* and the relationship between *PTPN6* and clinicopathologic characteristics in BC. *PTPN6* was highly expressed in BC compared with normal samples (a) and was correlated with grade, stage, T, and N (b–e). However, *PTPN6* was not associated with age, gender, and M (f–h).

**Figure 2:**
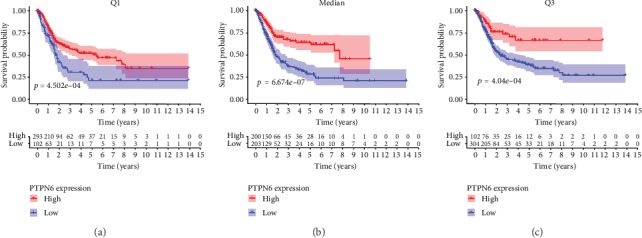
Survival analysis of *PTPN6* in BC. Kaplan-Meier curve revealed that low expression of *PTPN6* was significantly related to poor OS in BC (Q1, median, Q3; *P* = 4.502*e* − 04, 6.674*e*-07, 4.04*e*-04) (a–c).

The Wilcoxon signed-rank test was used to identify the expression level of *PTPN6* and the relationship between *PTPN6* and clinicopathologic characteristics in BC. The result showed that *PTPN6* was highly expressed in BC compared with normal samples (*P* = 5.19*e* − 05; Figure 1(a)) and was correlated with grade (low grade vs. high grade; *P* = 0.003), stage (stage I-II vs. stage III-IV; *P* = 4.75*e* − 06), T (T1-2 vs. T3-4; *P* = 1.685*e* − 04), and N (N0-1 vs. N2-3; *P* = 0.008) in BC (Figures 1(b)–1(e)). However, *PTPN6* was not associated with age (<69 vs. ≥69; *P* = 0.247), gender (female vs. male; *P* = 0.962), and M (M0 vs. M1; *P* = 0.495) (Figures 1(f)–1(h)). Survival analysis revealed that low expression of *PTPN6* was significantly related to poor OS (Q1, median, Q3; *P* = 4.502*e* − 04, 6.674*e*-07, 4.04*e*-04) (Figures 2(a)–2(c)). The univariate Cox regression analysis showed that age, stage, T, N, and *PTPN6* were related to the OS of BC patients (Table 2 A). However, multivariate Cox regression analysis indicated that *PTPN6* was not an independent prognostic factor for BC (Table 2 B).

### 3.3. Identification of *PTPN6* Coexpression Genes and Construction of a Prognostic Nomogram

**Figure 3:**
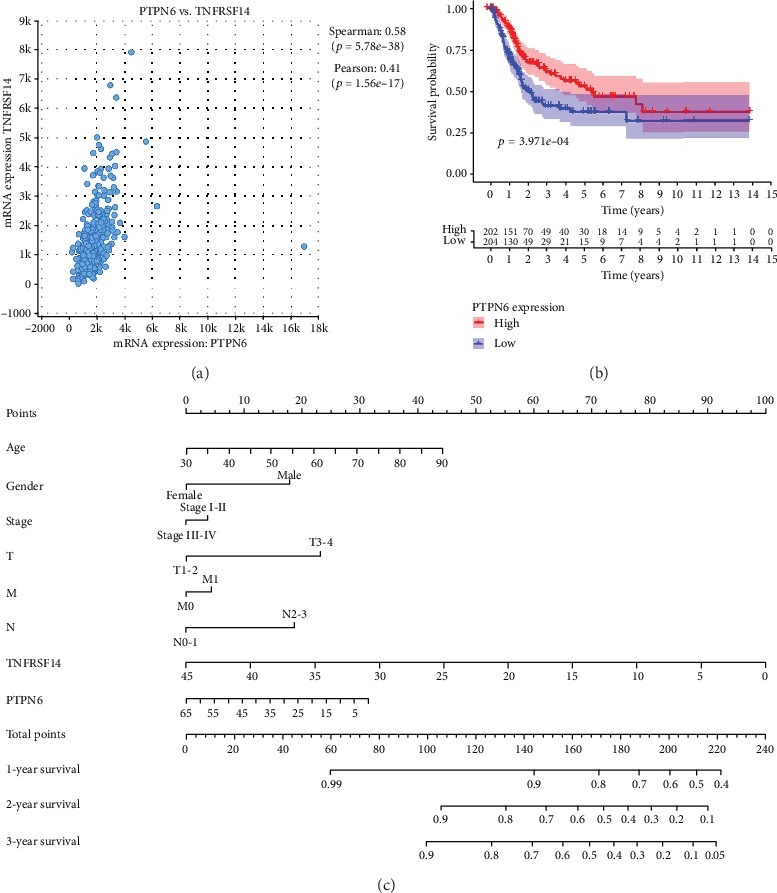
Coexpression analysis showed that *PTPN6* and *TNFRSF14* have a close correlation in BC (a). Survival analysis showed that *TNFRSF14* was associated with the OS of BC patients (b). Prognostic nomogram with clinicopathologic characteristics and gene expression (*PTPN6*, *TNFRSF14*) for BC. The nomogram could superiorly predict 1-, 2-, and 3-year OS of BC patients (c).

To better predict the prognosis of BC patients, coexpression analysis and a prognostic nomogram were constructed. According to the coexpression analysis of *PTPN6* in TCGA BC, we select *TNFRSF14* as the most relevant gene of *PTPN6* in BC (Figure 3(a)). Survival analysis showed that *TNFRSF14* was associated with the OS of BC patients (*P* = 3.971*e* − 04, Figure 3(b)). A nomogram by combing clinical factors (age, gender, stage, T, M, and N) and genes expression (*PTPN6*, *TNFRSF14*) values was established. The result revealed that the new prognostic nomogram could superiorly predict 1-, 2-, and 3-year survival outcomes of BC patients (Figure 3(c)).

### 3.4. GSEA Identified *PTPN6*-Related Signaling Pathways in BC

**Figure 4:**
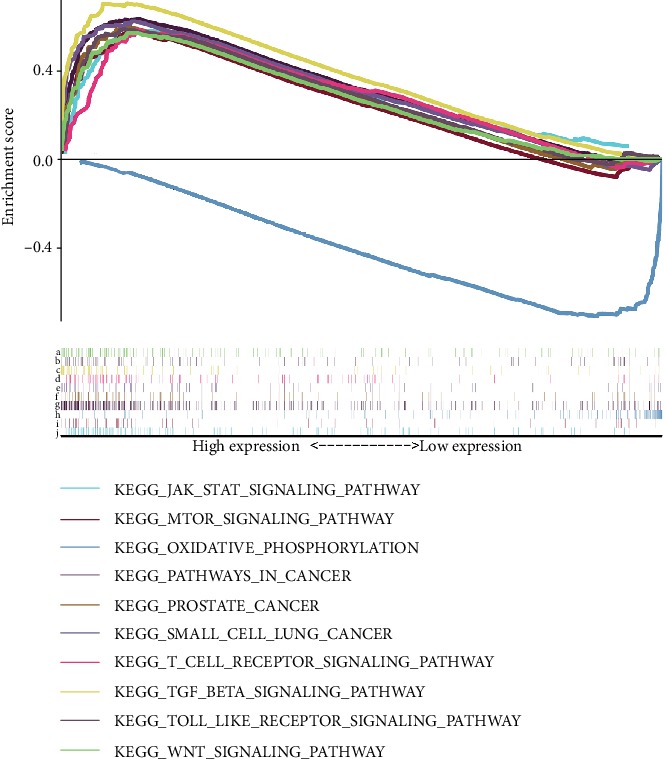
Enrichment plots from GSEA. GSEA results revealing that Wnt signaling, Toll-like receptor signaling pathway, TGF-beta signaling pathway, T cell receptor signaling pathway, small cell lung cancer, prostate cancer, pathways in cancer, oxidative phosphorylation, mTOR signaling pathway, and JAK-STAT signaling pathway are differentially enriched in *PTPN6* high expression phenotype (a–j).

To identify differentially activated signaling pathways in BC, we performed Gene Set Enrichment Analysis (GSEA) between low and high *PTPN6* expression datasets. GSEA reveals significant differences (FDR < 0.05, NOM *P* val < 0.05) in enrichment of MSigDB Collection (c2.cp.biocarta and h.all. v6.1. symbols). According to their normalized enrichment score (NES), the most significantly enriched signaling pathways were selected. The results showed that pathways in cancer, TGF-beta signaling pathway, JAK-STAT signaling pathway, Wnt signaling, Toll-like receptor signaling pathway, small cell lung cancer, prostate cancer, mTOR signaling pathway, oxidative phosphorylation, and T cell receptor signaling pathway are differentially enriched in *PTPN6* high expression phenotype (Figure 4 and Table 3).

### 3.5. Correlation Analysis between *PTPN6* Expression and Immune Cell Infiltration in BC

**Figure 5:**
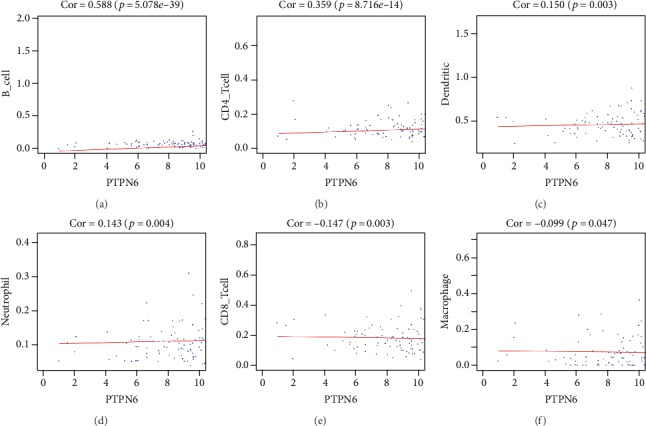
The relationship between *PTPN6* expression values and infiltration abundances of six types of immune cells. The results revealed that *PTPN6* was positively related to the infiltration of B cells, CD4+ T cells, dendritic cells, and neutrophils and negatively related to CD8+ T cells and macrophages (a–f).

We estimated the relationship between the abundance of six types of tumor-infiltrating immune cells (B cells, CD4+ T cells, CD8+ T cells, neutrophils, macrophages, and dendritic cells) and *PTPN6* expression values in BC. The results revealed that *PTPN6* was positively related to the infiltration of B cells (*P* = 5.078*e* − 39), CD4+ T cells (*P* = 8.716*e* − 14), and dendritic cells (*P* = 0.003), neutrophils (*P* = 0.004) and negatively related to CD8+ T cells (*P* = 0.003) and macrophages (*P* = 0.047) (Figures 5(a)–5(f)).

## 4. Discussion

BC is one of the most common human malignancies, and its pathogenesis is complex, involving a large number of genes expression, dysfunctions, and changes in multiple signaling pathways. Although many prognostic markers for BC have been identified, few have been applied broadly to make patient-specific decisions. Thus, screening of new potential prognostic markers for BC is still warranted. To our knowledge, no previous reports showed the prognostic value of *PTPN6* in BC. In the present study, we identified that *PTPN6* was significantly overexpressed in BC by using RNA-seq data from the TCGA database. The treatment strategies for BC patients depend largely on clinicopathological characteristics. Thus, we analyzed the relationship between PTPN6 and clinical factors and found that *PTPN6* was associated with grade, stage, T, and N. Survival analysis showed that *PTPN6* was closely correlated with the OS of BC patients. Previous studies have also shown that *PTPN6* can serve as a prognostic factor in cancers, such as neuroblastoma and peripheral T cell lymphomas [12–14]. Coexpression analysis indicated that *PTPN6* and *TNFRSF14* have a close correlation in BC. Zhu and Lu revealed that low expression level of *TNFRSF14* was associated with the poor survival outcomes in BC [15]. Furthermore, a nomogram by combing clinical factors and genes expression (*PTPN6*, *TNFRSF14*) values was established to predict the prognosis of BC patients.

To further investigate the potential molecular mechanisms of *PTPN6*, which associated with the survival outcomes of BC, we firstly performed GSEA. GSEA indicated that pathways in cancer, TGF-beta signaling pathway, JAK-STAT signaling pathway, Wnt signaling, Toll-like receptor signaling pathway, small cell lung cancer, prostate cancer, mTOR signaling pathway, oxidative phosphorylation, and T cell receptor signaling pathway are differentially enriched in *PTPN6* high expression phenotype. Wen et al. showed that *PTPN6* can inhibit the activation of JAK/STAT, NF-*κ*B, and AKT signaling pathways to inhibit the progression of hepatocellular carcinoma [5]. *PTPN6* can suppress growth and increase apoptosis in prostate cancer cells, which indicated that *PTPN6* may be a novel therapeutic target in prostate cancer [16]. In addition, a previous study revealed that *PTPN6* played an important role in antitumor immunity [17]. Therefore, we further analyzed the relationship between *PTPN6* and immune cell infiltration in the tumor environment. The results revealed that *PTPN6* was positively related to the infiltration of B cells, CD4+ T cells, dendritic cells, and neutrophils and negatively related to CD8+ T cells and macrophages. Watson et al. showed that the absence of *PTPN6* can control tumor growth by enhancing the ability of adoptively transferred CD8(+) T cells [18]. Previous studies also revealed that *PTPN6* played multiple roles in immune cells, such as the survival time of neutrophils, the activation of B cells, and the activation and survival time of T cells [19–22]. The number and type of immune cell infiltration in the tumor microenvironment was closely related to the occurrence and progression of BC. Therefore, *PTPN6* can influence the prognosis of BC patients by regulating a variety of cancer-related signaling pathways and remodeling the immune microenvironment of a tumor.

There are several limitations to the present study. First, as a retrospective study, our research still has a bias due to heterogeneity, although almost all the clinical factors in BC cohorts available from the public database have been included. Second, the study only provides preliminary bioinformatics evidence to understanding the significance of *PTPN6* in BC. Therefore, further experimental studies are needed in the future to explore the potential effect and mechanism of *PTPN6* in the prognosis of BC.

In conclusion, we identified that *PTPN6* may be a novel prognostic biomarker in BC based on the TCGA database. Further clinical trials are needed to confirm our observations and mechanisms underlying the prognostic value of *PTPN6* in BC also deserve further experimental exploration.

## Figures and Tables

**Table 1:** TCGA bladder cancer patient characteristics.

Clinical characteristics		Total (412)	%
Age at diagnosis		69 (34-90)	
Gender	Female	108	26.21
	Male	304	73.79
Histologic grade	High grade	388	94.17
	Low grade	21	5.1
Stage	I	2	0.49
	II	131	31.8
	III	141	34.22
	IV	136	33.01
T	T0	1	0.24
	T1	3	0.73
	T2	120	29.13
	T3	196	47.57
	T4	59	14.32
M	M0	196	47.57
	M1	21	5.1
N	N0	239	58.01
	N1	44	10.68
	N2	75	18.2
	N3	8	1.94

**Table 2:** Associations with overall survival and clinicopathologic characteristics in TCGA BC patients using univariate (a) and multivariate Cox regression (b) analysis.

Clinicopathologic variable	HR (95% CI)	*P* value
A		
Age	1.03 (1.00-1.06)	0.039
Gender	0.63 (0.36-1.10)	0.106
Stage	1.78 (1.24-2.54)	0.007
T	1.69 (1.15-2.50)	0.008
M	2.12 (0.76-5.88)	0.15
N	1.55 (1.18-2.03)	0.002
PTPN6	0.59 (0.38-0.91)	0.016
B		
Age	1.02 (0.99-1.05)	0.147
Gender	0.57 (0.31-1.02)	0.059
Stage	1.18 (0.59-2.38)	0.642
T	1.33 (0.81-2.20)	0.259
M	1.17 (0.37-3.69)	0.788
N	1.22 (0.73-2.04)	0.459
PTPN6	0.64 (0.40-1.02)	0.06

**Table 3:** GSEA identified *PTPN6*-related signaling pathways in BC.

MSigDB collection	Gene set name	NES	NOM *P* val	FDR *q* val
c2.cp.biocarta.v6.1.symbols.gmt	PATHWAYS_IN_CANCER	2.407	<0.0001	<0.0001
h.all.v6.1.symblos.gmt	TGF_BETA_SIGNALING_PATHWAY	2.403	<0.0001	<0.0001
	JAK_STAT_SIGNALING_PATHWAY	2.192	<0.0001	4.75*E*-04
	WNT_SIGNALING_PATHWAY	2.129	<0.0001	0.0015
	TOLL_LIKE_RECEPTOR_SIGNALING_PATHWAY	2.083	<0.0001	0.0018
	SMALL_CELL_LUNG_CANCER	2.054	<0.0001	0.0021
	PROSTATE_CANCER	2.050	<0.0001	0.0021
	OXIDATIVE_PHOSPHORYLATION	-2.052	0.006	0.0171
	T_CELL_RECEPTOR_SIGNALING_PATHWAY	1.922	0.012	0.0073
	MTOR_SIGNALING_PATHWAY	1.914	0.0061	0.0075

## Data Availability

The data used to support the findings of this study is included in the article, and the data are available from the corresponding author upon request.
